# Global Disparities in Cognitive and Functional Aging: A Cross-Sectional Study Across Countries

**DOI:** 10.1093/geroni/igaf122.260

**Published:** 2025-12-31

**Authors:** You Wu, Jie Shen, Marie-Theres Huemer, Magdalena Opazo Breton, Yuan Ma

**Affiliations:** Harvard T.H. Chan School of Public Health, Boston, Massachusetts, United States; Harvard T.H. Chan School of Public Health, Boston, Massachusetts, United States; Helmholtz Zentrum München, German Research Center for Environmental Health, Neuherberg, Bayern, Germany; University of Nottingham, Nottingham, England, United Kingdom; Harvard. T.H. Chan School of Public Health, Boston, Massachusetts, United States

## Abstract

With rapid population aging, understanding disparities in cognitive and functional aging between individuals and across countries is crucial for global healthy longevity. Our study described cognitive and physical function among nationally representative older adults (aged 65 and older) from China, England, India, Mexico, and USA, using data from 15,430 participants (53.7% women) in the Harmonized Cognitive Assessment Protocol (HCAP) and parent cohort studies. Cognitive function was assessed using harmonized cognitive function factors. Physical function was measured by the Activities of Daily Living scale (ADLs). We further explored how individual- and country-level characteristics may explain these variances using multi-level modelling. We observed that older adults in England and USA maintained better cognitive function than their peers in China, India, and Mexico throughout later life. The average cognitive function of older adults in China, India, and Mexico was equivalent to those approximately 16-25 years older in England and USA. England and USA also had a higher declining rate in cognitive function across age groups. Similar cross-country disparities were observed for ADLs, with an increasing proportion of dependency with age in all countries. The individual-level (age, sex, education, and multimorbidity) and country-level covariates (socio-demographics and universal health coverage) jointly explained 14.5% within-country variances and 43.8% between-country variances in cognitive function, but only 2.7% and 0.3% for ADLs. Our findings showed a wide global disparity in cognitive and functional aging, while individual- and country-specific characteristics partly explained these variances. Future studies may analyze how these characteristics interact over time to influence healthy aging.

